# MicroRNA Expression Varies according to Glucose Tolerance, Measurement Platform, and Biological Source

**DOI:** 10.1155/2017/1080157

**Published:** 2017-04-26

**Authors:** S. Dias, S. Hemmings, C. Muller, J. Louw, C. Pheiffer

**Affiliations:** ^1^Biomedical Research and Innovation Platform (BRIP), South African Medical Research Council, Tygerberg, South Africa; ^2^Division of Molecular Biology and Human Genetics, Faculty of Medicine and Health Sciences, Stellenbosch University, Tygerberg, South Africa; ^3^Department of Psychiatry, Faculty of Medicine and Health Sciences, Stellenbosch University, Tygerberg, South Africa; ^4^Division of Medical Physiology, Faculty of Health Sciences, Stellenbosch University, Tygerberg 7505, South Africa; ^5^Department of Biochemistry and Microbiology, University of Zululand, Kwa-Dlangezwa 3886, South Africa

## Abstract

Dysregulated microRNA (miRNA) expression is observed during type 2 diabetes (T2D), although the consistency of miRNA expression across measurement platform and biological source is uncertain. Here we report miRNA profiling in the whole blood and serum of South African women with different levels of glucose tolerance, using next generation sequencing (NGS) and quantitative real time PCR (qRT-PCR). Whole blood-derived miRNAs from women with newly diagnosed T2D (*n* = 4), impaired glucose tolerance (IGT) (*n* = 4), and normal glucose tolerance (NGT) (*n* = 4) were subjected to NGS, whereafter transcript levels of selected miRNAs were quantified in the whole blood and serum of these women using qRT-PCR. Of the five significantly differentially expressed miRNAs identified by NGS, only the directional increase of miR-27b in women with IGT compared to NGT was confirmed in whole blood and serum, using qRT-PCR. Functional enrichment of miR-27b gene targets identified biological pathways associated with glucose transport and insulin regulation. In conclusion, this study showed poor correlation in miRNA expression profiled using NGS and qRT-PCR and in whole blood and serum. The consistent increased expression of miR-27b in women with IGT compared to NGT across measurement platform and biological source holds potential as a biomarker for risk stratification in our population.

## 1. Introduction

Diabetes mellitus is one of the leading causes of morbidity and mortality in the world, with an estimated global prevalence of 9% reported in 2014 [1] and 1.5 million deaths directly attributed to diabetes in 2012 [2]. Type 2 diabetes mellitus (T2D), which accounts for about 90% of diabetes cases, is a complex metabolic disorder characterised by chronic hyperglycemia due to decreased insulin action and increased pancreatic beta- (*β*-) cell dysfunction [3, 4]. Initially, *β*-cells compensate for declined insulin action by increasing insulin secretion so as to maintain glucose homeostasis. This state is characterised by hyperinsulinemia and impaired fasting glucose (IFG) and/or impaired glucose tolerance (IGT) [5]. Progressive deterioration in insulin sensitivity results in hyperglycemia and *β*-cell dysfunction.

MicroRNAs (miRNAs) are small, noncoding RNAs, approximately 22 nucleotides in length, that modulate gene expression by silencing messenger RNA (mRNA) expression through translational inhibition or mRNA degradation [6]. Their dysregulation has been demonstrated during many chronic diseases such as cancer, T2D, and neurological disorders [7, 8], bolstering interest in miRNAs as molecular markers underlying disease pathophysiology. Recently, miRNAs expressed within blood cells or as cell-free miRNAs in plasma/serum have attracted considerable interest as biomarkers of disease due to their stability [9–11]. Studies have reported that these circulating miRNAs reflect tissue expression and function [12], arguing that, in addition to their potential as biomarkers, these miRNAs could play a role in disease pathophysiology.

A single miRNA can regulate the expression of over 200 mRNA targets [13]; thus profiling these master regulators of gene expression allows the unravelling of the complex network of molecular pathways associated with T2D. Indeed, the number of studies profiling miRNAs during T2D has increased considerably over the last few years, with a variety of measurement platforms and biological sources used [14–16]. Chen et al. (2009) reported considerable variation between miRNA expressions using qRT-PCR arrays and microarrays, with higher variation observed in low abundance miRNAs [15], Wang et al. (2012) reported differential miRNA expression in serum and plasma samples [14], while Pritchard et al. (2012) reported a correlation between circulating miRNAs and their expression in one or more blood cell types [16].

In this study, we report miRNA profiling across glucose tolerance, measurement platform, and biological source. miRNAs from the whole blood of South African women with different levels of glucose tolerance were compared using NGS and qRT-PCR, whereafter miRNA expression in whole blood and serum was compared using qRT-PCR. Messenger RNA (mRNA) target prediction and functional enrichment analysis was conducted for miR-27b, which showed consistent expression across glucose tolerance, measurement platform, and biological source.

## 2. Materials and Methods

### 2.1. Study Population

Subjects were women of mixed ethnic ancestry who resided in the Western Cape Province of South Africa. Individuals of mixed ethnicity have Khoisan, black, white, and Asian ancestry and constitute 8.9% of the South African population and 48.8% of the population in the Western Cape Province, where the study was conducted [17]. A prevalence survey conducted between 2008 and 2009 in a mixed ethnic ancestry community in the Western Cape reported an age-adjusted T2D prevalence of 26.3% in this population [18]. Women were not on any form of medication for health-related issues including T2D. All women provided informed voluntary consent, and all procedures were conducted according to the Declaration of Helsinki and approved by the ethics committee of the South African Medical Research Council (EC010-5/2013) and the Human Research Ethics Committee of Stellenbosch University (S15/04/095).

### 2.2. Clinical Data

Blood pressure, body weight, and height were measured using standardised procedures. Blood glucose concentrations were measured using the 75 g oral glucose tolerance test (OGTT) as recommended by the World Health Organisation (WHO) after an overnight fast of 8 to 10 hrs. Glycated haemoglobin A1c (HbA1c), insulin, glucagon, C-peptide, cholesterol, and lipid concentrations were measured by an accredited laboratory (Pathcare Laboratories, Cape Town, South Africa). Blood for miRNA profiling from whole blood was collected in PAXgene® Blood RNA Tubes (PreAnalytix, Qiagen, Feldbachstrasse, Switzerland), while blood for miRNA profiling in serum was collected in BD Vacutainer® SST™ Tubes (Vacutainer, BD, Woodlands, South Africa). Subjects were classified as T2D, IGT, or NGT according to WHO criteria [1].

### 2.3. RNA Isolation

miRNAs from whole blood were isolated from blood collected in PAXgene® Blood RNA Tubes, using the PAXgene blood miRNA kit (Qiagen, Hilden, Germany), according to the manufacturer's instructions. Cell-free circulating miRNAs were isolated from serum using the miRNeasy Serum/Plasma kit (Qiagen), according to the manufacturer's instructions. To correct for technical variation during cell-free miRNA isolation, 3.5 *μ*l of MiRNeasy serum/plasma spike-in control (*Caenorhabditis elegans* miR-39) (1.6 × 10^8^ copies/*μ*l working solution) was added to the RNA during the extraction process. The quantity and quality of RNA were assessed using the NanoDrop ND-1000 instrument (Nanodrop Technologies, Wilmington, USA) and the Bioanalyser 2100 system (Agilent Technologies, CA, USA) using the Agilent RNA 6000 Nano and small RNA kits (Agilent Technologies), respectively, according to the manufacturer's instructions.

### 2.4. MicroRNA Sequencing

Whole blood-derived miRNAs were profiled by NGS on an Illumina Hiseq 2000 instrument (Illumina Inc., San Diego, USA) using the Illumina TruSeq Rapid SBS preparation protocol (Arraystar Inc., Rockville, USA). Complementary DNA (cDNA) was prepared as follows: Total RNA was separated by polyacrylamide gel electrophoresis and bands corresponding to the size of the miRNAs (~15–35 nt) were excised from the gel. Thereafter, 3′ and 5′ small RNA adapters were ligated to the size selected RNA molecules by T4 RNA ligase and then reverse-transcribed to generate cDNA. The cDNA library was run on a polyacrylamide gel and the band corresponding to the molecular size of miRNA fragments with ligated adapters (~135–155 bp) was excised from the gel for subsequent sequencing. The DNA size, purity, and quantification of the cDNA library were measured using a DNA 1000 kit on a 2100 Bioanalyser (Agilent Technologies). The cDNA libraries were diluted to a final concentration of 8 pM and cluster generation was performed on the Illumina cBot using the TruSeq Rapid SR cluster kit, according to manufacturer's instructions. Single stranded DNA molecules were captured on Illumina flow cells, amplified in situ as clusters, and sequenced for 36 cycles on the Illumina Hiseq 2000, according to the manufacturer's instructions. After sequencing images were generated, image analysis and base calling were performed using the Off-Line Basecaller software (OLB v1.8.0). All trimmed reads greater than or equal to 15 nt in length, with zero or one base pair mismatch, were aligned to the latest human reference miRNA precursor set (Sanger miRBase 19) (http://mirbase.org) using Novoalign software (v2.07.11). In order to characterise the isomiR variability, sequences that matched the miRNA precursor in the mature miRNA region ±4 nt (no more than 1 mismatch) were accepted as mature miRNA isomiRs. To estimate the relative expression level of miRNAs and to compare expression patterns between groups, the expression of each unique sequence among the reads in the sample was normalised (as reads per million) against the total number of reads produced for the sample. Differentially expressed miRNAs between two groups were analysed using the two tailed, homoscedastic* t*-test.

### 2.5. Quantitative Real Time PCR

For whole blood-derived miRNAs, qRT-PCR was performed using TaqMan assays (Life Technologies, Carlsbad, USA), according to the manufacturer's protocol. Briefly, 10 ng of miRNA-enriched total RNA was reverse-transcribed with miRNA-specific, stem-loop primers (Supplementary Table S1 in Supplementary Material available online at https://doi.org/10.1155/2017/1080157) using the TaqMan® MicroRNA Reverse Transcription Kit (Life Technologies). Thereafter, 1.33 *μ*l of cDNA was amplified using 1 *μ*l of TaqMan miRNA probes and 10 *μ*l of TaqMan® Universal PCR Master Mix II (with AmpErase®) in a total reaction volume of 20 *μ*l. The thermal cycler (Applied Biosystems 7500) was set at 50°C for 2 mins and 95°C for 10 min, followed by 40 cycles of 95°C for 15 s and 60°C for 1 min, using a 96-well plate, according to manufacturer's instructions.

For cell-free miRNA profiling in serum, miScript miRNA assays (Supplementary Table S1) (Qiagen, Hilden, Germany) were used. Briefly, 90 ng of miRNA-enriched total RNA was reverse-transcribed into cDNA using the miScript II RT kit (Qiagen). The cDNA was diluted with 200 *μ*l of RNAse-free water and then used as a template in qRT-PCR. Each reaction was performed in a final volume of 10 *μ*l containing 1 *μ*l of diluted cDNA, 5 *μ*l of miScript 2x SYBR Green, 1 *μ*l of miScript 10x primer assay, and 1 *μ*l of miScript 10x universal primer as the reverse primer. The thermal cycler was set at 95°C for 5 mins, followed by 40 cycles of 94°C for 15 s, 55°C for 30 s, and 70°C for 34 s, according to manufacturer's instructions.

Relative expression levels were calculated using the delta delta Ct (2^−ΔΔCT^) method, with the average expression of RNU6B, miR-454, and miR-425 used as an endogenous control for whole blood-derived miRNA expression, or miR-425 and miR-39 as an endogenous and internal spike-in control for cell-free miRNA expression, respectively. Normfinder, a mathematical model of gene expression, was used to confirm the stable expression of the endogenous reference miRNAs in the dataset [19].

### 2.6. MiR-27b Target Prediction Analysis

Messenger RNA gene targets were identified using TargetScan (v6.2) (http://www.targetscan.org) [20], Miranda (2010-11-01) [21], and Microcosm v5 (http://www.ebi.ac.uk/enright-srv/microcosm/htdocs/targets/v5/). MiRNA gene targets common to all three programs were filtered using the Venny tool (Venny v2.0.2) (http://bioinfogp.cnb.csic.es) [22].

### 2.7. MiR-27b Functional Enrichment Analysis

Commonly predicted gene targets were subjected to functional analysis using gene ontology (GO) (TopGO v4.2) grouping categories (Biological Process, Cellular Components and Molecular Processes) and Kyoto Encyclopedia of Genes and Genomes (KEGG) pathway analysis (Arraystar Inc., Rockville, USA). Statistical significance for GO terms was calculated using Fisher's exact test. The significance of KEGG pathways was determined using the EASE-score, Fisher* p* value, and the Hypergeometric test adjusted for the false discovery rate (FDR) [23].

### 2.8. Statistical Analysis

Quantitative real time PCR data are presented as the mean ± standard error of the mean (SEM). Statistical significance was determined using the one-way analysis of variance (ANOVA), followed by Tukey post hoc and an unpaired* t*-test where appropriate (GraphPad Prism v5.02) (GraphPad Software, San Diego California, USA). A *p* ≤ 0.05 was considered statistically significant. Statistical analysis of sequencing and bioinformatics results are discussed in the relevant sections.

## 3. Results

### 3.1. Clinical Characteristics of Participants

The clinical parameters of participants are presented in Table 1. All subjects were between 46 and 48 years of age, female, obese (BMI > 30 kg/m^2^), and of mixed ethnic ancestry. As expected, the 2 hr OGTT values were different between groups (13.4 ± 0.7 versus 8.9 ± 0.3 versus 5.6 ± 0.3 mmol l^−1^, *p* < 0.0001) for T2D, IGT, and NGT, respectively. Fasting plasma glucose concentrations were higher in T2D compared to NGT individuals (6.7 ± 0.5 versus 5.1 ± 0.1 mmol l^−1^, *p* < 0.05), while HbA1c values were higher in T2D compared to IGT individuals (6.5 ± 0.2 versus 5.6 ± 0.2%, *p* < 0.05) and in T2D compared to NGT individuals (6.5 ± 0.2 versus 5.4 ± 0.07%, *p* < 0.01). Blood pressure, fasting and 2 hr insulin and C-peptide concentrations, and glucagon and triglyceride concentrations were increased in individuals with IGT compared to both NGT and T2D individuals, although not statistically significant (Table 1).

### 3.2. MicroRNA Expression Profiling Using Illumina Sequencing

A total of 263 miRNAs identified by Illumina sequencing were mapped to the human reference miRNA precursor set (Sanger miRBase 19). Of these, five miRNAs (miR-27b, miR-98, miR-143, miR-21, and miR-379) were significantly differentially expressed between T2D, IGT, and NGT groups (Table 2).

### 3.3. Quantitative Real Time PCR of MicroRNAs in Whole Blood

**Figure 1:**
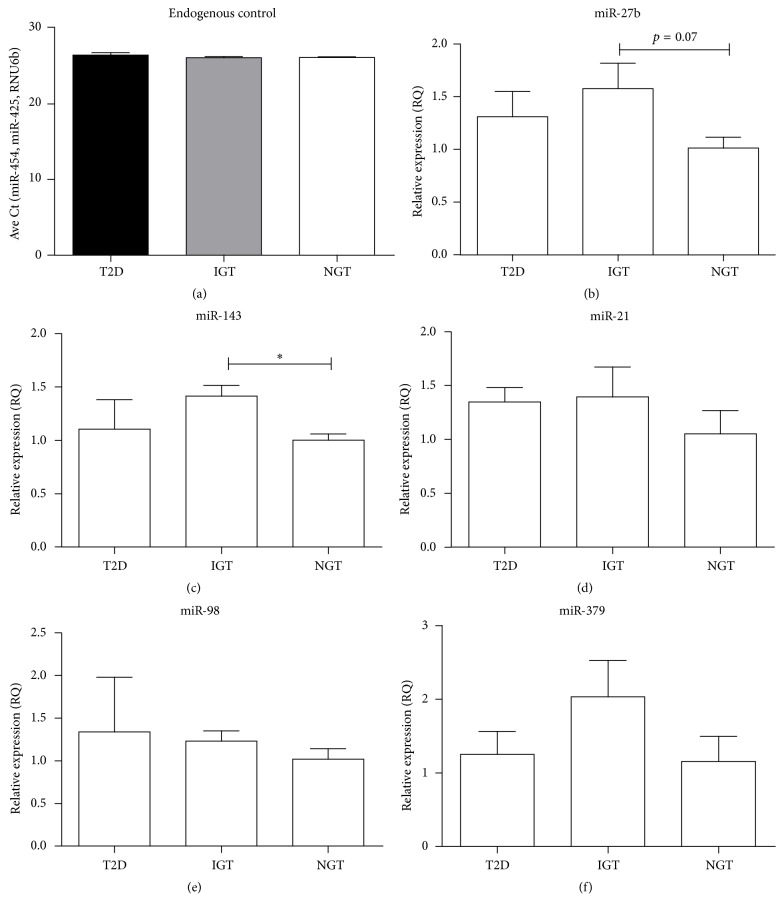
Quantitative real time PCR of differentially expressed miRNAs identified by NGS. The average Ct of endogenous controls, miR454, miR425, and RNU6b (a), and relative expression levels of miR-27b (b), miR-143 (c), miR-21 (d), miR-98 (e), and miR-379 (f) in T2D (*n* = 4), IGT (*n* = 4), and NGT (*n* = 4). Data are presented as the mean ± error of the mean (SEM). ^*∗*^*p* < 0.05.

To explore the correlation of miRNA expression in NGS and qRT-PCR, the expression of miR-27b, miR-98, miR-143, miR-21, and miR-379 was measured in the whole blood of the same set of women using qRT-PCR. No difference in the miRNA expression of the endogenous controls (miR-454, miR-425, and RNU6b) was observed (Figure 1(a)). Of the five miRNAs tested, qRT-PCR results for miR-27b confirmed sequencing results (1.55-fold; *p* = 0.07), while no correlation was observed for miR-21, miR-379, and miR-98. MiR-143 was significantly upregulated in IGT compared to NGT (1.4-fold, *p* ≤ 0.01) in contrast to sequencing, where a significant downregulation in IGT compared to T2D was observed (Figure 1).

### 3.4. Quantitative Real Time PCR of MicroRNAs in Serum

**Figure 2:**
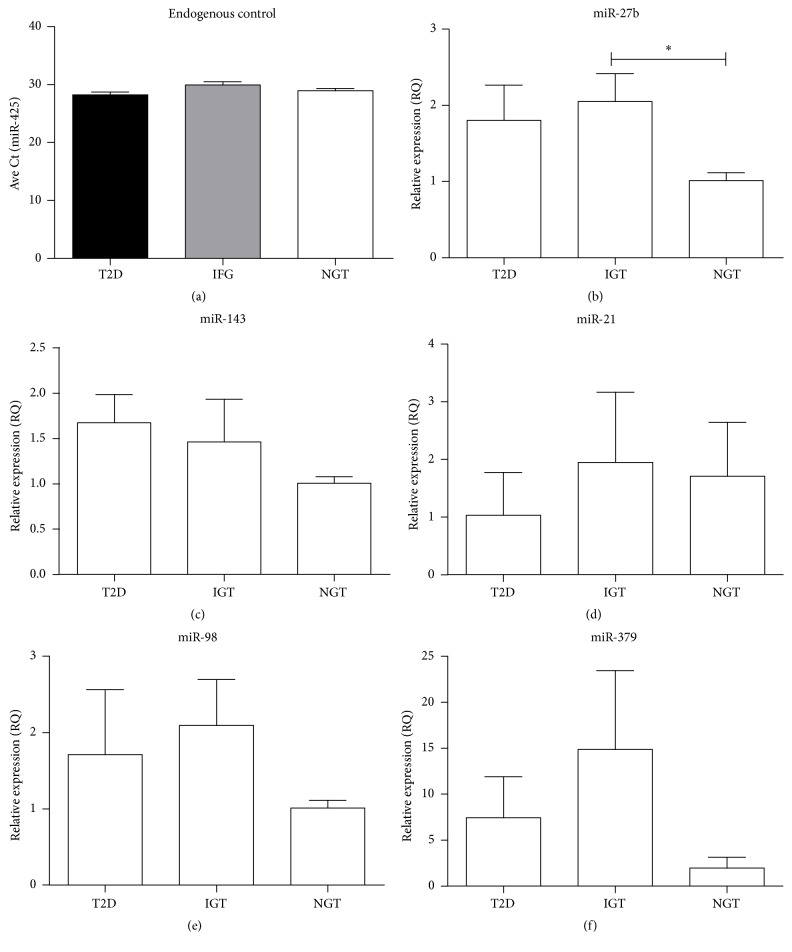
Quantitative real time PCR of differentially expressed miRNAs in serum. The average Ct of endogenous control miR425 (a) and relative expression changes of miR-27b (b), miR-143 (c), miR-21 (d), miR-98 (e), and miR-379 (f) from serum were analysed using quantitative real time PCR in T2D (*n* = 4), IGT (*n* = 4), and NGT (*n* = 4). Data are presented as the mean ± error of the mean (SEM). ^*∗*^*p* < 0.05.

To investigate miRNA correlation between whole blood and serum, the expression of miR-27b, miR-98, miR-143, miR-21, and miR-379 was quantified in the serum of the same set of women using qRT-PCR. As expected, no difference in the expression of the endogenous (miR-425) (Figure 2(a)) and the internal spike-in control (miR-39) was observed between T2D, IGT, and NGT. MiR-27b was upregulated by 2.0-fold (*p* ≤ 0.05) during IGT compared to NGT. Although miR-21, miR-379, miR-98, and miR-143 were also detected in serum, none of these were differentially expressed between the groups (Figure 2). Of the five miRNAs investigated, only the directional consistent expression of miR-27b in the IGT and NGT groups observed in whole blood was confirmed.

### 3.5. Target Prediction of miR-27b

**Figure 3:**
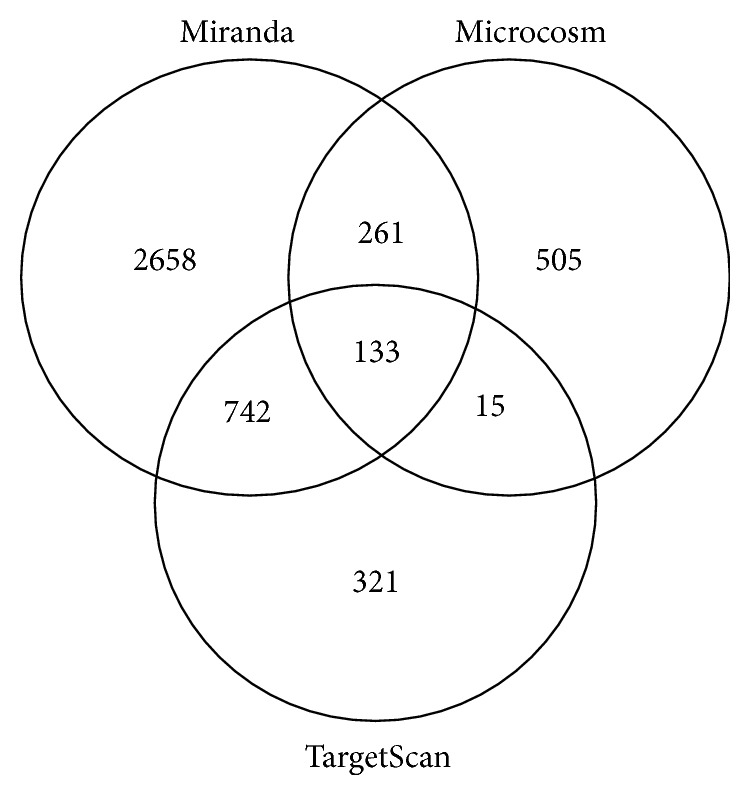
Venn diagram showing mRNA targets for miR-27b predicted using TargetScan, Miranda, and Microcosm. More than 3000 mRNA targets for miR-27b were predicted by each program. Of these, 742 mRNA targets were commonly predicted by TargetScan and Miranda, 261 mRNA targets were commonly predicted by Miranda and Microcosm, 15 mRNA targets were commonly predicted by Microcosm and TargetScan, and 133 mRNA targets were commonly predicted by TargetScan, Miranda, and Microcosm.

Over 3000 putative mRNA targets were predicted for miR-27b using TargetScan, Miranda, and Microcosm, of which 133 gene targets were commonly predicted by all three programmes (Figure 3). Moreover, 742 mRNA targets were commonly predicted by TargetScan and Miranda, 261 mRNA targets were commonly predicted by Miranda and Microcosm, and 15 mRNA targets were commonly predicted by Microcosm and TargetScan.

### 3.6. Functional Enrichment Analysis of miR-27b

**Figure 4:**
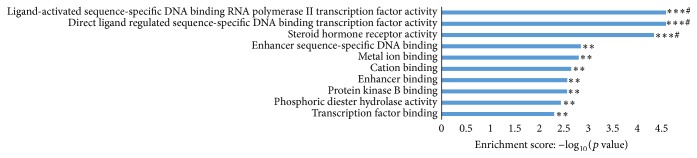
Top ten GO terms enriched by miR-27b gene targets. Functional enrichment of miR-27b targets was conducted using TopGO. Data are presented as enriched scores expressed as −log_10_(*p* value). ^*∗∗∗*^*p* value ≤ 0.001, ^*∗∗*^*p* value ≤ 0.01, and ^#^FDR ≤ 0.01.

**Figure 5:**
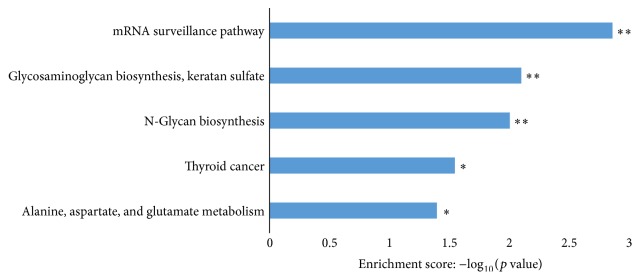
KEGG pathway analysis of miR-27b gene targets. MiR-27b gene targets were mapped to KEGG pathways using TopGO. Data are presented as enriched scores expressed as −log_10_(*p* value). ^*∗∗*^Fisher *p* value ≤ 0.01, ^*∗*^*p* value ≤ 0.05.

Functional enrichment analysis identified 464 GO terms enriched by miR-27b gene targets, categorized into 41 molecular functions, 404 biological processes, and 11 cellular components (Supplementary Table S2), with the top 10 GO terms illustrated in Figure 4. Three GO terms, (1) direct ligand regulated sequence-specific DNA binding transcription factor activity, (2) ligand-activated sequence-specific DNA binding RNA polymerase II transcription factor activity, and (3) steroid hormone receptor activity, were statistically significant with *p* < 0.001, FDR ≤ 0.01, and >12-fold enrichment (Supplementary Table S2). Five significantly enriched signalling pathways (Fisher *p* value ≤ 0.05) (Supplementary Table S3) were identified. These pathways include the (1) mRNA surveillance, (2) glycosaminoglycan biosynthesis, keratan sulfate, (3) N-glycan biosynthesis, (4) thyroid cancer, and (5) alanine, aspartate, and glutamate metabolism pathways (Figure 5).

## 4. Discussion

Dysregulated miRNA expression has been reported in several tissues associated with T2D, including the pancreas, liver, skeletal muscle, and adipose tissue [24, 25], suggesting an important role for these master gene regulators in the molecular pathophysiology of T2D. Moreover, their potential as biomarkers for T2D has increased studies profiling miRNAs using different measurement platforms and biological sources. In this study, we report that miRNA expression in South African women of mixed ethnic ancestry, who constitute a large proportion of the T2D disease burden in South Africa [17, 18], varies according to glucose tolerance, measurement platform, and biological source.

NGS identified 263 miRNAs in the whole blood of South African women of mixed ethnic ancestry, of which five were significantly differentially expressed between T2D, IGT, and NGT groups. Analysis of these miRNAs in the same samples by qRT-PCR confirmed the directional increased expression of miR-27b during IGT compared to NGT only. Interestingly, compared to the other four miRNAs (miR-98, miR-143, miR-21, and miR-379), miR-27b exhibited the lowest fold-change between the groups. The fold-change in miR-27b expression was however associated with the lowest *p* value and FDR, suggesting that in our study statistical significance, rather than fold-change, increased the likelihood of true biological difference, as evidenced by consistent expression between NGS and qRT-PCR. Although qRT-PCR confirmed the expression of all miRNAs, discordance in their expression across glucose tolerance groups was observed. The inconsistencies between NGS and qRT-PCR could be due to technological differences between the platforms. For example, NGS quantifies miRNAs according to sequence read counts, while qRT-PCR measures transcript abundance relative to an endogenous control [26]. Other technical aspects include template quality which affects reverse transcription and PCR efficiency [27, 28].

The potential of miRNAs as biomarkers for T2D is widely reported. Studies have reported differential expression of miRNAs in whole blood, peripheral blood mononuclear cells (PBMCs), serum, and plasma. Zampetaki et al. (2010) identified a panel of 10 plasma miRNAs (miR-20b, miR-21, miR-24, miR-15a, miR-126, miR-191, miR-197, miR-223, miR-320, and miR-486) that were able to distinguish between T2D patients and NGT individuals [29]. Another study identified seven serum-derived miRNAs (miR-9, miR-29a, miR-30d, miR34a, miR-124a, miR146a, and miR375) that were increased in T2D patients compared to NGT individuals [10]. Moreover, Balasubramanyum et al. (2010) observed decreased expression of miR-146a in PBMCs, of patients with T2D, and reported that miR-146a was associated with insulin resistance and poor glycaemic control during T2D [30].

Arguments for and against the use of whole blood for biomarker discovery have been reported. Compared to serum and plasma, whole blood is relatively easy to collect and has higher RNA content, facilitating more accurate and reliable miRNA expression measurements. However, whole blood contains different blood cells that may confound biomarker discovery [31]. For example, whole blood consists of a mixture of erythrocytes, lymphocytes, and platelets, which vary between individuals and in response to disease stages, thus contributing to the heterogeneity observed between samples [16]. The use of serum may decrease these confounding variables, possibly allowing the detection of specific disease-related miRNA expression profiles [31]. Studies have profiled miRNAs within blood cells or as cell-free miRNAs in serum [32], although limited studies have compared miRNA expression in serum and whole blood in T2D, IGT, and NGT individuals. In this study, only the consistent disease-specific expression of one of the five miRNAs differentially expressed in whole blood was replicated in serum, thus demonstrating a poor correlation of miRNA expression in whole blood and serum.

Due to the cross-sectional nature of the study, we were not able to explore the role of miR-27b in the molecular pathophysiology of T2D. Women were characterised as IGT or T2D according to their blood glucose levels, which do not reflect the complexity and heterogeneity of individual pathophysiology and the different disease states possibly present [33]. Moreover, women of mixed ethnic ancestry, a highly heterogeneous genetic group, were used in this study, further contributing to individual heterogeneity. In addition, miRNAs are epigenetic factors known to be affected by a variety of environmental factors, which are not known for these women. The relevance of miR-27b to molecular physiology should be investigated in a longitudinal study design where the same individuals are followed up from NGT to T2D.

Using NGS and qRT-PCR, this study showed that whole blood and serum-derived miR-27b correlated with IGT. To our knowledge this is the first study directly associating miR-27b with IGT. Others have reported increased expression of miR-27b during cancer [34], cardiovascular disease [35], adipogenesis [36], atherosclerosis [37], and type 1 diabetes and diabetic nephropathy [38], and although recent studies have suggested a role for miR-27b during obesity and T2D [36, 39–42], studies have been unable to demonstrate differential expression of miR-27b during T2D, IGT, and NGT [43]. Thus, using a NGS and global miRNA profiling approach rather than measuring the expression of miRNAs identified by others [10, 44, 45] could lead to the identification of population-specific miRNAs, an important consideration for biomarker discovery. Although the results of this study are limited due to the small sample size, the lack of independent validation, and the analysis of only one gender, our results support future longitudinal studies in larger samples sizes to explore the potential of miR-27b as a biomarker for IGT in our population.

MiR-27b mRNA targets were enriched in biological processes and pathways related to cell communication and signal transduction, cell differentiation, cell development, regulation of apoptotic processes, and transcription, consistent with the function of miRNAs in normal development and cellular homeostasis. Three of the top 10 GO terms enriched for biological processes were associated with glucose transport and the synthesis, secretion, and action of insulin [46–48], suggesting a critical regulatory role for miR-27b in insulin-dependent glucose homeostasis. Moreover, gene targets for miR-27b were enriched in the protein kinase B (PKB) signalling and N-glycan biosynthesis pathways that have been associated with T2D [48–50].

## 5. Conclusion

In conclusion, this study shows that miRNA expression varies according to glucose tolerance, measurement platform, and biological source and adds to the growing body of evidence demonstrating altered miRNA expression during T2D. Interestingly, this study showed increased expression of miR-27b in the whole blood and serum of women with IGT compared to NGT, supporting its potential as a biomarker for IGT in our population; however, longitudinal studies in larger sample sizes are required to explore this theory.

## Supplementary Material

Supplementary Table S1 lists the assay names of differentially expressed miRNAs identified by next generation sequencing, and the mature miRNA sequences that were used to design miRNA-specific stem-loop primers and probes for miRNA profiling with quantitative real time PCR. Supplementary Table S2 lists the GO terms enriched for miR-27b gene targets, which are categorised into 41 molecular functions, 404 biological processes and 11 cellular components. The supplementary table lists: 1) GO term ID and name, 2) the total number of differentially expressed and background population genes associated with each GO term ID, 3) fold enrichment values, 4) p-values, 5) false discovery rate, 6) enrichment scores and 7) gene ratio values. Supplementary Table S3 lists the significantly enriched signalling pathways identified for miR-27b gene targets using KEGG pathway analysis. The table lists: 1) pathway ID, 2) definition of the pathway ID, 3) website for differentially expressed genes, 4) fisher p-value, 5) the total number of differentially expressed and background population genes associated with pathway ID, 6) false discovery rate, 7) enrichment scores and 8) gene ratio values.

## Figures and Tables

**Table 1:** Clinical parameters of study participants.

	T2D	IGT	NGT
*N*	4	4	4
Age (years)	46.8 ± 6.6	47.3 ± 6.9	46.3 ± 5.7
Gender	Female	Female	Female
Ethnicity	Mixed ancestry	Mixed ancestry	Mixed ancestry
BMI (kg/m^2^)	36.7 ± 3.1	38.6 ± 3.7	33.7 ± 1.8
2 hr OGTT (mmol l^−1^)	**13.4 ± 0.7** ^*γ*†^	**8.9 ± 0.3** ^*γ*Ψ^	**5.6 ± 0.3** ^†Ψ^
FPG (mmol l^−1^)	**6.7 ± 0.5** ^*∗*^	5.4 ± 0.3	**5.1 ± 0.1** ^*∗*^
HbA1c (%)	**6.5 ± 0.2** ^*∗γ*^	**5.6 ± 0.2** ^*∗*^	**5.4 ± 0.07** ^*γ*^
Blood pressure (mmHg):			
Systolic	113.7 ± 10.8	131.3 ± 5.9	118.5 ± 4.0
Diastolic	72.3 ± 8.7	83.3 ± 4.4	82.0 ± 3.4
Fasting insulin (ng ml^−1^)	23.6 ± 6.7	34.8 ± 6.3	21.6 ± 3.1
2 hr insulin (ng ml^−1^)	97.8 ± 28.4	264.4 ± 87.7	92.5 ± 23.7
Fasting C-peptide (ng ml^−1^)	2.7 ± 0.6	2.9 ± 0.4	2.7 ± 0.2
2 hr C-peptide (ng ml^−1^)	7.9 ± 1.8	11.1 ± 1.7	9.1 ± 0.8
Glucagon (pg ml^−1^)	91.0 ± 9.5	114.2 ± 16.9	79.6 ± 6.6
Cholesterol (mmol l^−1^)	5.5 ± 0.7	5.3 ± 0.3	5.4 ± 0.8
HDL (mmol l^−1^)	1.2 ± 0.1	1.2 ± 0.1	1.3 ± 0.03
LDL (mmol l^−1^)	3.8 ± 0.6	3.6 ± 0.3	3.6 ± 0.7
Triglycerides (mmol l^−1^)	1.2 ± 0.3	1.7 ± 0.3	1.1 ± 0.2

*N* = number of participants; BMI = body mass index; FPG = fasting plasma glucose; OGTT = oral glucose tolerance test; HbA1c = glycated haemoglobin A1c; 2 hr refers to a test performed 2 hrs after an oral glucose challenge. HDL = high-density lipoprotein; LDL = low-density lipoprotein.

^*∗*^ *p* ≤ 0.05; ^*γ*^*p* ≤ 0.01; ^†Ψ^*p* ≤ 0.001.

**Table 2:** Differentially expressed microRNAs identified by next generation sequencing.

Gene symbol	Mature miRNA ID	Disease group	Fold change	*p* value
MI0000100	hsa-miR-98-5p	T2D vs. IGT	−1.25	0.04
MI0000459	hsa-miR-143-3p	T2D vs. IGT	1.75	0.05
MI0000077	hsa-miR-21-3p	T2D vs. IGT	1.22	0.008
MI0000787	hsa-miR-379-5p	T2D vs. IGT	1.12	0.02
MI0000440	hsa-miR-27b-3p	IGT vs. NGT	1.15	0.003
MI0000100	hsa-miR-98-5p	IGT vs. NGT	−1.23	0.02

Positive values indicate miRNAs that were upregulated in the first group compared to the second group, while negative values indicate miRNAs that were downregulated in the first group compared to the second group. The 3p and 5p suffix indicate that the mature miRNA originated from either the 3′ or 5′ arm of the primary miRNA.
